# Orange peels magnetic activate carbon (MG-OPAC) composite formation for toxic chromium absorption from wastewater

**DOI:** 10.1038/s41598-023-30161-6

**Published:** 2023-02-28

**Authors:** Asmaa Khalil, Mohamed Salem, Safaa Ragab, Mika Sillanpää, Ahmed El Nemr

**Affiliations:** 1 Department of Chemistry, Faculty of Science, Tanta University, Tanta, Egypt; 2 Environment Divisions, National Institute of Oceanography and Fisheries (NIOF), Kayet Bey, Elanfoushy, Alexandria, Egypt; 3 Department of Chemical Engineering, School of Mining, Metallurgy and Chemical Engineering, University of Johannesburg, P. O. Box 17011, Doornfontein, 2028 South Africa; 4 Department of Applied Physics, Faculty of Science and Technology, Universiti Kebangsaan Malaysia, 43600 Bangi, Selangor Malaysia; 5 Zhejiang Rongsheng Environmental Protection Paper Co. LTD, NO.588 East Zhennan Road, Pinghu Economic Development Zone, Zhejiang, 314213 People’s Republic of China; 6 Department of Civil Engineering, University Centre for Research & Development, Chandigarh University, Gharuan, Mohali, Punjab India; 7 International Research Centre of Nanotechnology for Himalayan Sustainability (IRCNHS), Shoolini University, Solan, Himachal Pradesh 173212 India

**Keywords:** Chemical engineering, Environmental chemistry

## Abstract

This work prepared a composite of orange peels magnetic activated carbon (MG-OPAC). The prepared composite was categorized by X-ray diffraction (XRD), Fourier-transform infrared spectroscopy (FTIR), Brunauer–Emmett–Teller (BET), Energy-dispersive X-ray spectroscopy (EDX), Scanning Electron Microscopy (SEM) and vibrating-sample magnetometer (VSM) analyses. The MG-OPAC composite showed the surface area (155.09 m^2^/g), the total volume of pores (0.1768 cm^3^/g), and the mean diameter of pores (4.5604 nm). The saturation magnetization (Ms = 17.283 emu/g), remanence (Mr = 0.28999 emu/g) and coercivity (Hc = 13.714 G) were reported for the prepared MG-OPAC. Likewise, at room temperature, the MG-OPAC was in a super-paramagnetic state, which could be collected within 5 S (< 5 S) with an outside magnetic field. Influence of time of contact, absorbent dose, starting concentration of Cr^6+^ ions, and pH were tested to adjust the absorption process. The absorption behavior of MG-OPAC for hexavalent chromium was investigated by Langmuir (LIM), Freundlich (FIM) and Temkin (TIM) isotherm models (IMs). Applicability of LIM specifies that Cr^6+^ ions absorption procedure may be monolayer absorption. The maximum monolayer capacity (*Q*_m_) premeditated by LIM was 277.8 mg/g. Similarly, the absorption process was tested with different kinetic models like intraparticle diffusion (IPDM), pseudo-first-order (PFOM), Elovich (EM), pseudo-second-order (PSOM), and Film diffusion (FDM). The PSOM was best fitted to the experimental results of Cr^6+^ ions absorption with *R*^2^ ranging between 0.992 and 1.

## Introduction

Clean water is the most pressing issue confronting humanity, owing to the circumstance of the WHO considers water to be the most vital food product, owing to its importance to our health^1^. Access to safe and clean water is critical for ecosystems, living species, and humans. Due to population development, industrialization, and agricultural activity, existing clean water and freshwater sources are decreasing.

So, controlling water pollution has become increasingly important in recent years, making the treatment of industrial effluents a difficult problem in environmental research. Significant amounts of wastewater from home, industrial, and agricultural sources are still dumped into clean bodies of water. As a result, water contamination occurs, directly and indirectly affecting humans and natural ecosystems. Furthermore, most wastewater is discharged into the oceans, causing significant environmental damage in various ways^2–4^.

Wastewater contaminated with heavy metals like chromium, copper, arsenic, cadmium, zinc, mercury, nickel, and lead is one of the extremely remarkable environmental difficulties of this era. Heavy metals (HMs) removal is essential because they are harmful and carcinogenic elements that shouldn't be released into the environment directly^5–9^. As they leach into the surface and groundwater, they are absorbed by fishes and vegetables and then stored in the human body by the food chain, consequently causing acute or chronic diseases^10–12^. Chromium has two stable oxidation states in aqueous systems: trivalent and hexavalent. The trivalent form is necessary for life, while the hexavalent form is poisonous, mutagenic, and carcinogenic^13–15^. It can be absorbed through the skin and is a strong oxidant that is highly mobile in soil and water. The maximum amount of chromium that can be present in drinking water is 0.05 ppm for Cr^6+^ and 5 ppm for Cr^3+^, according to WHO standards^16,17^.

Hexavalent chromium is one of the most dangerous HM ions. It originated from leather tanning, electroplating, mining tailings, cement manufacturing, wood preservation, and other industries' effluents^18^. Also, it has been recorded among the top 20 pollutants on the Superfund Priority List of Hazardous Substances^19^.

For the treatment of wastewater, many techniques have been utilized to remove hexavalent chromium, such as adsorption^20,21^, reduction, ion exchange^22^, electrochemical processes^23^, precipitation^24^, and reverse osmosis^25,26^. Adsorption is thought to be a likely strategy because it is straightforward, simple to use, and effective at removing contaminants^27–29^. Several sorbents have been utilized to adsorb chromium from polluted waters, such as activated carbon (AC), sphagnum moss peat, fly ash and *Wollastonite*, and *Pinus* *sylvestris* bark^21^.

The adsorption behavior of AC is because of its extended surface area, highly established pore structure, and functional groups that are produced during the manufacturing process^30,31^. Because activated carbon is less expensive and easier to regenerate than more advanced purification products like reverse osmosis membranes, and ion exchange resins, it is a preferred method of treatment. The orange peel employed in this study often contains surface functional groups like amid, –OH, and –COOH. It also contains cellulose, hemicellulose, lignin, and pectin components. Activated carbon (AC) properties and forms of porosity, containing the surface chemistry, the size, shape, and distribution of the pores of the material, depending on the types of materials used, the activation techniques used, and the conditions in which they are activated^32,33^. Preparation and evaluation of bio-based magnetic AC have been reported as an effective adsorbent for the malachite green dye from water^34^. Magnetic activated carbon prepared from polyethylenimine and acorn shells as magnetic adsorbents were used, respectively, in the removal of uranium and methylene blue from water^35,36^. Kinetic and thermodynamic studies for rhodamine B removal by magnetic AC/CeO_2_ nanocomposite were investigated^37^. A collection of colorimetric sensing of heavy metals on metal-doped metal oxide nanocomposites has been reported^38^. Ultrasonic green synthesis method is reported for the preparation of zinc oxide nanoparticles loaded on activated carbon (AC) derived from coffee biomass^39^.

Both chemical and physical activation processes can be applied to create AC. Raw materials were carbonized at high temperatures in an inert atmosphere using the physical activation process, which was then activated using air, steam, or CO_2_^40^. In the chemical activation process, raw materials were soaked with activator (dehydrating chemical agents) such as H_3_PO_4_^41^, H_2_SO_4_^42^, NaOH^43^, KOH^44^, ZnCl_2_^45^, then carbonized in an inert atmosphere (with inert gases like nitrogen, argon) at a certain temperature. With a large surface area, clearly defined porous materials, excellent yields, and the greatest adsorption capacity, activated carbon employing ZnCl2 as a chemical activator was the best^46,47^.

Powdered AC (PAC) and granular AC (GAC) are the two main AC used to treat wastewater. Most researchers used GAC for the adsorption of pollutants from the water as it is easy to be separated (isolated) from the bulk fluid by classification or screening; however, PAC requires less contact time and lower capital cost than GAC. Thus, magnetic PAC (PMAC), a promising composite material that can be isolated from water using an external magnetic field, is created by mixing PAC with magnetic iron oxide nanoparticles (magnetic separators).

Consequently, this work aims to form a composite magnetic orange peel activated carbon (MG-OPAC) using ZnCl_2_ as a chemical activator and investigate as an absorbent for Cr^6+^ ions adsorption from water. The obtained MG-OPAC was categorized by XRD, surface area analysis, FT-IR, EDX, SEM and VSM. Magnetite nanoparticles are important for removing the MG-OPAC adsorbent from the adsorption solution after the completion of the curing process. The influence of process variables on the absorption process, like pH, contact time, absorbent mass, and starting concentration of Cr^6+^ ions, were investigated. Numerous kinetic and isotherm models were investigated to achieve a better consideration of the absorption process.

## Materials and methods

### Chemicals and materials

Orange peels were gathered from a local market in Alexandria, Egypt, cleaned with distilled water (DW), and dried at 50 °C for 24 h. The dried peels were crushed in a mixer and put away until they were needed. Stock solution of Cr^6+^ ions was organized by dissolving 2.83 g of K_2_Cr_2_O_7_ in 1 L DW. Potassium dichromate (K_2_Cr_2_O_7_, M.W 294.185 g, assay 99.5%) and Ferric nitrate anhydrous (Fe(NO_3_)_3_, M.W. 241.86 g, assay 98%) were gotten from ADWIC, El-Nasr Chemical Company, Egypt. BDH Chemicals LTD provided the 1,5-diphenylcarbazide used as a substance for Cr^6+^ ions, while Universal Fine Chemicals PVT-LTD in Mumbai, India provided ZnCl_2_ (M.W.136.30 g, assay 99.5%). From SD Fine-Chem. Limited (SD FCL), we got HCl (M.W. 36.46 g, test 30–34%). For the synthesis of magnetite Fe_3_O_4_, ferrous sulphate (FeSO_4_^.^7H_2_O, M.W. 278.01 g, assay 98.5%) was acquired from Alpha Chemika in India. None of the compounds was further purified before usage.

### Synthesis of AC from orange peels (OPAC)

Orange peels were cleaned with DW and dried for 20 h at 50 °C. The dried peels were pulverized in a blender before being activated with ZnCl_2_ in a 1:2 (W/W) ratio at 105 °C for 24 h. After that, it was held at 700 °C for 1 h with a nitrogen flow of 50 mL/min in a tubular furnace (T.F. Nabertherm B180 (RT 50/250/13)). It created OPAC in powder form. After being cooled to ambient temperature, the activated carbon was refluxed with 1N HCl for 2 h to eliminate the alkali and then washed with DW to achieve a neutral pH. It was then dried for four hours at 105 °C.

### Preparation of orange peels magnetic AC (MG-OPAC)

One gram of activated carbon (AC) was suspended in 500 mL of a solution comprising 3.5 g (8.66 mmoL) Fe(No_3_)_3_ and 1.3 g (4.33 mmoL) FeSO_4_·7H_2_O to create magnetic activated carbon (MG-OPAC). The reaction was made at 50 °C with strong stirring for 1 h after the solution was sonicated (40 W, 200 kHz) for 10 min to precipitate the iron oxide. Next, NaOH aqueous solution was introduced drop-wise into the suspension until pH was elevated to 11–12. The precipitate was periodically rinsed with DW until pH was neutral before being removed from the water dispersion by an external magnetic field (or by filtration). After drying, the MG-OPAC composite was stored in a bottle until usage.

### Artificial wastewater

By dissolving 2.827 g of K_2_Cr_2_O_7_ in DW and diluting it to 1 L, the stock solution of (1.0 g L1) Cr^6+^ ions was created. This standard solution was properly diluted to create the Cr^6+^ ions working solution. Using a pH metre and 0.1 M NaOH or HCl, the liquids' pH was changed. The 1,5-diphenylcarbazide technique was used to spectrophotometrically determine the Cr^6+^ ions concentration^48^. The 1.5-diphenylcarbazide reagent was made by combining 0.1 g of the compound with 6 mL of methanol, 1 mL of concentrated sulfuric acid, and stirring with a magnetic stirrer. The mixture was then diluted to 1 L with DW. To make the standard curve, concentrations between 10 and 150 mg/L were created from the stock solution.

### Sample characterization

Using a surface area and pore analyzer, N_2_ adsorption/desorption isotherms at 77.4 K and a relative pressure (*P*/*P*°) range of 0.001–1 were used to measure the specific surface area (*S*_BET_) (BELSORP—Mini II, BEL Japan, Inc). The *S*_BET_, total volume of pores (*V*_T_), and mean diameter of pores (*D*_P_) of the produced MG-OPAC were calculated by the Brunauer–Emmett–Teller (BET) model^49^. Additionally, using the BELSORP analysis programme software, the *t*-plot approach was used to measure the micropore surface area (*S*_mi_) and micropore volume (*V*_mi_). Utilizing a Bruker VERTEX70 with a platinum ATR model V-100 in the wave number range of 400–4000 cm^–1^, FTIR spectroscopy was used to recognize the functional groups existent in OPAC, orange peels magnetic activated carbon (MG-OPAC), and MG-OPAC composite after removal. Utilizing SEM, the produced composite's morphology was examined. In addition, EDX was employed to pinpoint the precise elements present on the sample surfaces under study by using SEM QUANTA 250 linked to EDX. The crystalline characteristics of MG-OPAC were categorized by XRD using a Panalytica X-Ray Diffractometer with Cu K_α_ radiation (k = 0.15406 nm) in the scanning range 2*Ɵ* (0–90). Vibrating sample magnetometers (VSM), made by VSM Lakeshore type 7410 in the USA, were applied to test the magnetic properties of MG-OPAC at room temperature.

### Batch adsorption investigation

The impacts of numerous significant parameters, including the amount of adsorbent, contact time, and pH values between adsorbent and adsorbate, were examined in this experiment using the batch adsorption approach. 100 mL of water containing Cr^6+^ ions was agitated with absorbent MG-OPAC at 25 °C and 200 rpm to conduct batch adsorption tests. After the desired time of contact, the flask was removed, centrifuged, and the residual concentration of Cr^6+^ ions was measured spectrophotometrically through the 1,5-diphenylcarbazide procedure (50 µL superannuated + 2 mL reagent). On the absorption of Cr^6+^ ions, the effects of MG-OPAC mass (0.1–0.4 g/L), contact period (15–180 min), and starting adsorbate Cr^6+^ ions concentration (100–300 mg/L) were examined^50–52^. Equations (1, 2) were used to compute the adsorbent's *q*_e_ adsorption capacity (mg/g).

1 $${q}_{e}=\frac{\left({C}_{i}-{\mathrm{C}}_{e}\right)V}{W},$$

2 $$R\%=\frac{{C}_{i}-{C}_{e}}{{C}_{i}}\times 100,$$

where *C*_i_ (mg L^–1^) is starting concentration of Cr^6+^ ions, *C*_e_ (mg L^–1^) is the residual concentration of Cr^6+^ ions gotten at the equilibrium state, *V* (L) is the solution volume, *R*% is the percentage of removal, and *W* (g) is the weight of adsorbent.

#### Author statement for the use of plants

In this study, Experimental research and field studies on plant material (Orange peels), including the collection of plant waste material, complies with relevant institutional, national, and international guidelines and legislation.

## Results and discussion

### Materials characterization

#### BET surface area

**Figure 1:**
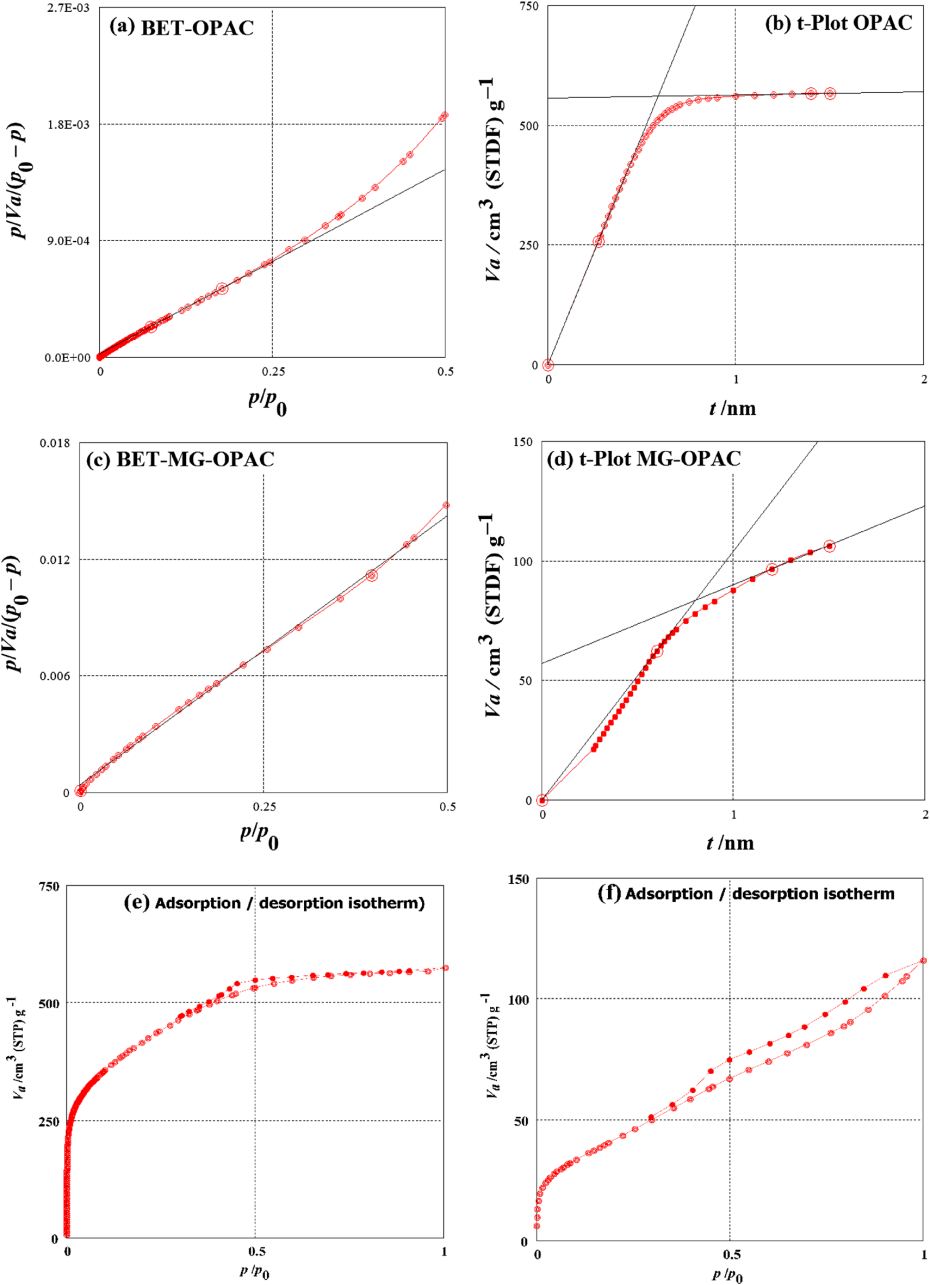
(**a**) BET surface area curve of OPAC, (**b**) t-Plot surface area curve of OPAC, (**c**) BET surface area curve of MG-OPAC, (**d**) t-Plot surface area curve of MG-OPAC, (**e**) Adsorption–desorption isotherm of OPAC, (**f**) Adsorption–desorption isotherm of MG-OPAC.

The *S*_BET_, total volume of pores (*V*_T_), and mean diameter of pores (*D*_P_) of the produced MG-OPAC were calculated by the Brunauer–Emmett–Teller (BET) model. The impact of the temperature of carbonization on the preparation of OPAC was studied at temperatures fluctuating from 700 to 900 °C using BET and *t*-plot models (Table 1). As shown in Table 1, the higher values obtained for surface area and pore volume of OPAC are 1512.5 m^2^/g and 0.8854 cm^3^/g, respectively, at carbonization temperature 700 °C (Fig. 1a,b), and then decrease with increasing temperature to 900 °C. Heat shrinking of pores is the cause of the surface area, and the volume of pores decreases with rising temperature^53^.

**Table 1:** Influence of carbonization temperature on the volume of pores and surface area of OPAC.

Model	Temp. (°C)	BET			*t*-plot	
Sample		*S*_BET_ (m^2^ g)	*V*_T_ (cm^3^/g)	*D*_P_ (nm)	*S*_mi_ (m^2^/g)	*V*_mi_ (cm^3^/g)
OPAC	700	1512.5	0.8854	2.3415	1474.0	0.8624
	800	1472.1	0.8411	2.2855	1444.1	0.8147
	900	1411.6	0.9506	2.6935	1323.9	0.9211

For prepared MG-OPAC, a smaller S_BET_ (155.09 m^2^ g^–1^), *V*_T_ (0.1768 cm^3^/g) and *D*_P_ (4.5604 nm) (Fig. 1c,d) were obtained due to the incorporation of iron oxide (Fe_3_O_4_) over the porous surface of OPAC. Figure 1c explains the N_2_ adsorption–desorption isotherms of fabricated MG-OPAC. The OPAC sample isotherm (Fig. 1e) demonstrated step type I isotherms (Micropores), while the MG-OPAC sample isotherm (Fig. 1f) demonstrated step type IV isotherms (Mesopores) following the classification of IUPAC^54,55^. The knee formation of OPAC shows monolayer-coated micropores. The isotherm IV of MG-OPAC suggests that absorbate gas fills pores at relatively low pressures, and the observed plateau suggests multilayer adsorption through the pores at moderate pressures. The existence of capillary condensation in the mesopores (Type IV isotherm) in MG-OPAC, on the other hand, was made clear by an increase in absorbed volume at high relative pressure.

#### XRD investigation

**Figure 2:**
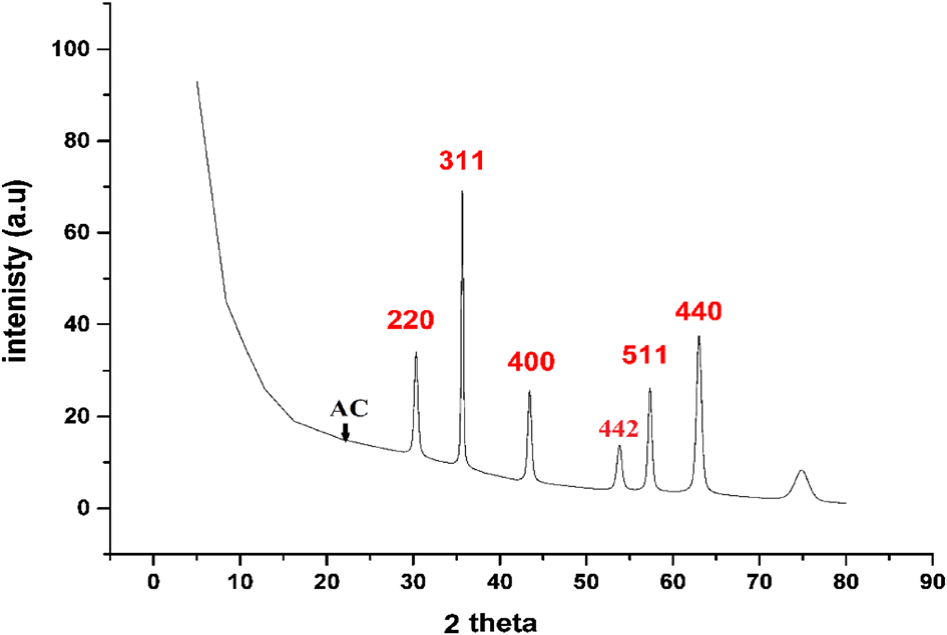
XRD pattern of the prepared MG-OPAC.

XRD was used to distinguish the crystalline properties of MG-OPAC using X-ray Defractommeter (panalytica) with Cu Kα radiation (k = 0.15406 nm) in the scanning range 2*Ɵ* (0–90). Found XRD pattern of the MG-OPAC is presented in Fig. 2 (according to card NO 00-001-1111). The observed bands at 2*Ɵ* = 30.1°, 35.5°, 43°, 57.2° and 62.7° link to 220, 311, 400, 511, and 440 faces of Fe_3_O_4_. The composite synthesis of Fe_3_O_4_ and activated carbon was validated by the acquired XRD decoration^56^. Extreme crystalline was formed at 2*Ɵ* = 35.5°. The low surface area of MG-OPAC can be explained via the XRD sharp peak. The XRD data can prove the low specific surface area in the case of MG-OPAC.

#### FTIR investigation

**Figure 3:**
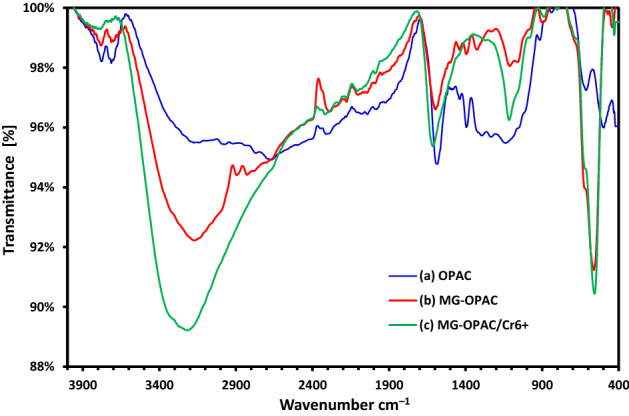
FTIR investigation of (**a**) OPAC, (**b**) MG-OPAC, (**c**) Cr^6+^ ions absorbed on MG-OPAC.

FTIR spectroscopy was used to recognize the functional groups existent in OPAC, orange peels magnetic activated carbon (MG-OPAC), and MG-OPAC composite after removal process of Cr^6+^ ions at the wavenumber range 400–4000 cm^−1^. Figure 3a illustrates FTIR spectrum of OPAC as a broad peak at 3775.4–3715.9 cm^–1^ is assigned to O–H bonding^57^. This O–H group occurs because of the existence of H_2_O molecules probably caused by semi-dried samples during the analysis. The peak at 2322.6, 1583.64 cm^–1^ correlated to C–H in the CH_3_ group and C=C aromatic ring, respectively^58^. The peak at 1393.4 cm^–1^ is because of O–H bending, which indicates the presence of oxygen in the sample. The peak at 1137.8 cm^–1^ corresponds to C–O stretching of COOH, phenol, alcohol, ether and ester^59^. The peak at 644.87 cm^–1^ is due to C–C stretching vibration. The spectrum in Fig. 3b illustrates the existence of a new intense peak at 563.9 cm^–1^, which could be assigned to M–O peak, possibly indicating the interaction between iron and oxygen in the samples^60^. In Fig. 3c peak due to C=C aromatic ring, C–O stretching and M–O peak was shifted to (1616.19, 1115.77, 557.85) cm^–1^ and these FTIR spectra changes approve the binding of Cr^6+^ ions with active groups existent in the MG-OPAC adsorbent.

#### VSM investigation

**Figure 4:**
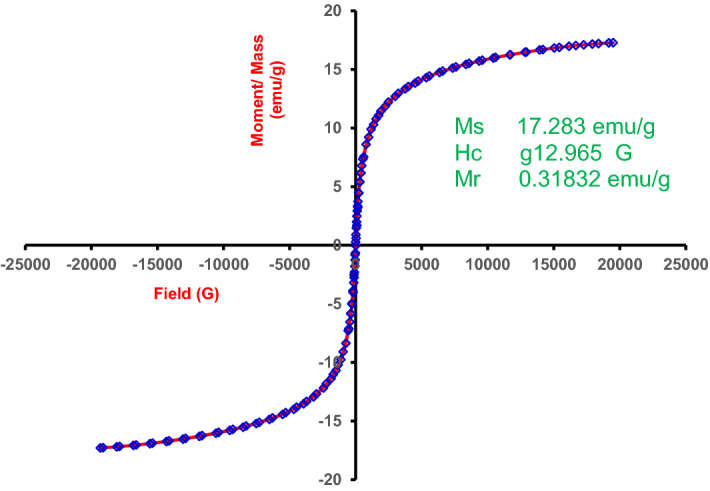
Magnetic hysteresis loop of nanocomposite MG-OPAC_._

The magnetic hysteresis loop of MG-OPAC (OPAC/Fe_3_O_4_), as depicted in Fig. 4, showed almost no coercivity and remanence, demonstrating the composite's usual paramagnetic behaviour. The values of the coercive force (Hc), remanance (Mr), and saturation magnetization (Ms) were determined to be 17.283 emu/g, 0.31832 emu/g, and 12.965 G, respectively. According to the ratio, Mr/Ms (0.02), the MG-OPAC was in a super-paramagnetic state at ambient temperature due to the low Mr value of less than 25%. The composite may be obtained with an external magnetic field in less than 5 s, according to inset Fig. 4^61,62^.

#### SEM and EDX analysis

**Figure 5:**
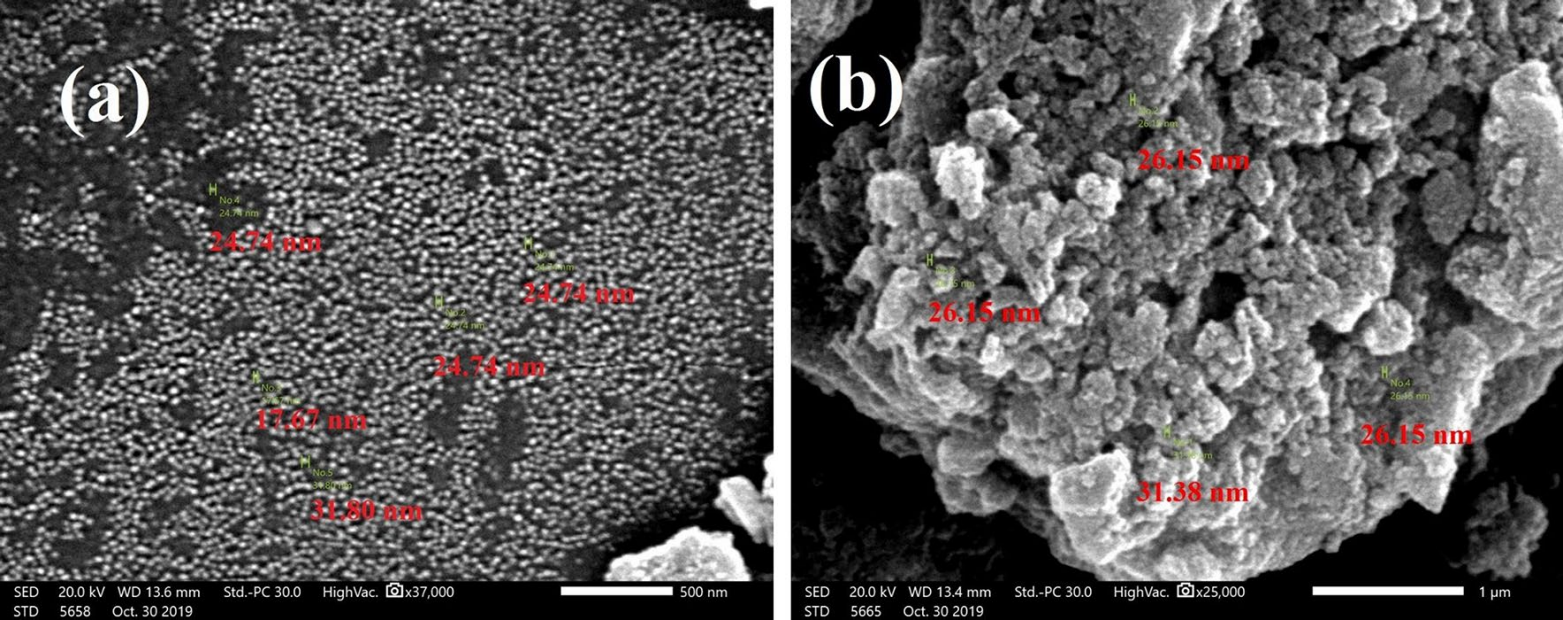
SEM micrograph of (**a**) OPAC under magnification of 37,000, (**b**) MG-OPAC under magnification of 25,000.

The surface images of the manufacturing of OPAC and MG-OPAC were shown using scanning electron microscopy (SEM) analysis. The exact elements on the surface of the examined samples were identified by Energy-dispersive X-ray spectroscopy (EDX). SEM and EDX images were recorded by using QUANTA 250. Figure 5b illustrates that Fe_3_O_4_ deposited onto the surface of OPAC. The SEM image of MG-OPAC (Fig. 5b) shows that most of the micropores in OPAC (Fig. 5a) were filled with Fe_3_O_4_ particles which explains the low specific surface area reported by BET for MG-OPAC. As seen from Fig. 5a, the surface of OPAC has porous textures, and the OPAC pores were opened due to the activation with ZnCl_2_. From Fig. 5b, it is possible to recognize that the surface of MG-OPAC has a homogenous appearance after being filled partially with Fe_3_O_4_ particles. Also, the magnetic molecules were held more into the pores because of the magnetite particles on the OPAC surface during the process^34^.

**Figure 6:**
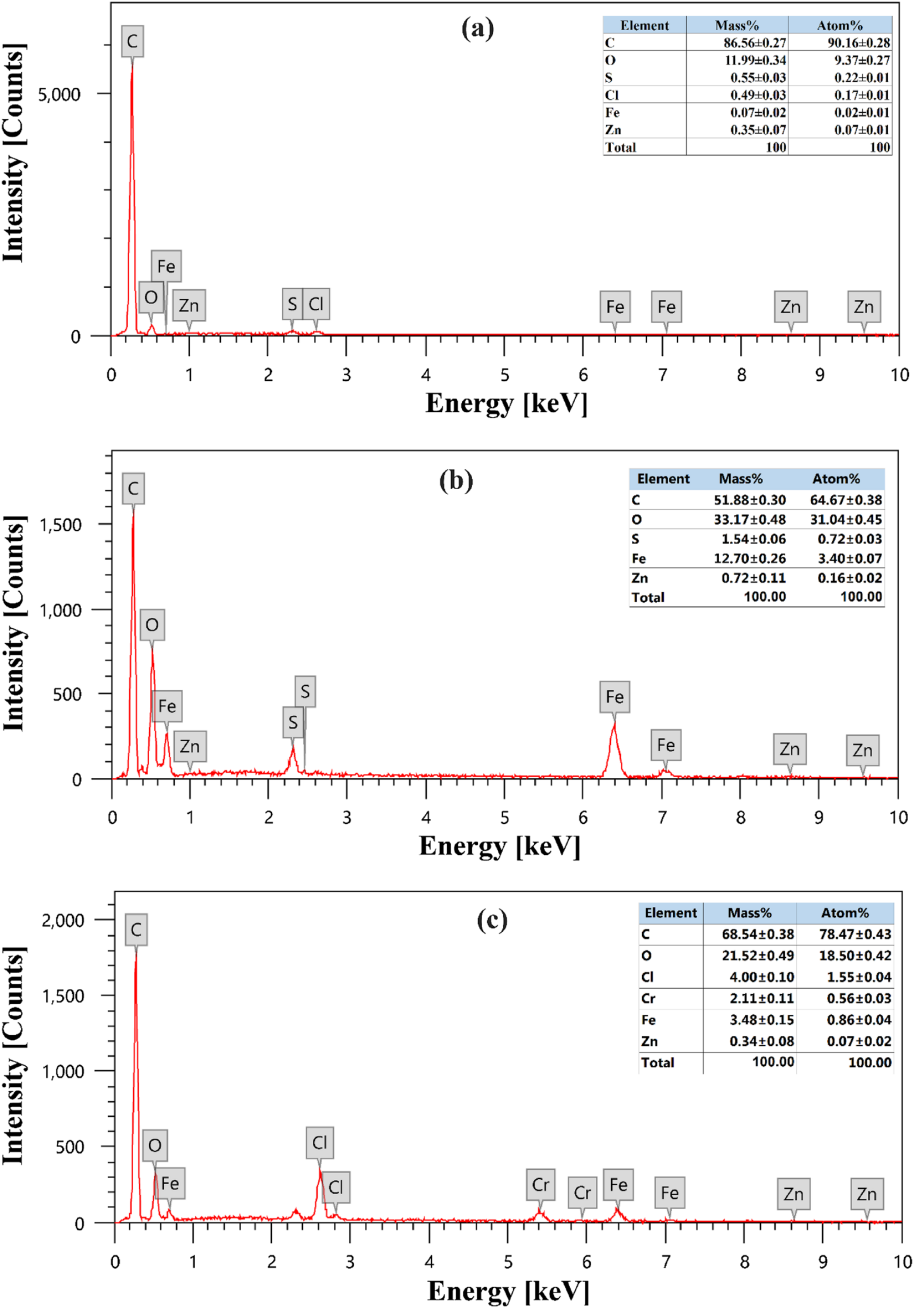
EDX investigation of (**a**) OPAC, (**b**) MG-OPAC composite, (**c**) Cr^6+^ ions absorbed on MG-OPAC.

EDX spectrum for OPAC, MG-OPAC composite, and Cr^6+^ ions adsorbed on MG-OPAC composite exists in Fig. 6. Figure 6a displays the EDX spectrum of OPAC, which exposed that the content of carbon was 86.30%, followed by oxygen 11.99%. The use of ZnCl_2_ in the chemical activation preparation of OPAC was blamed for the presence of zinc and chlorine in the sample. The EDX spectrum of MG-OPAC is depicted in Fig. 6b, and revealing five major elements: carbon, sulphur, oxygen, zinc, and iron. The presence of sulfur was ascribed to using FeO_4_S in preparation for MG-OPAC. However, the presence of iron was thought to have resulted from iron oxide that had formed on the surface of the OPAC during the magnetization. Chromium has been adsorbed on the surface of MG-OPAC, as shown in Fig. 6c.

### Absorption investigation

#### Influence of pH on removal efficiency

**Figure 7:**
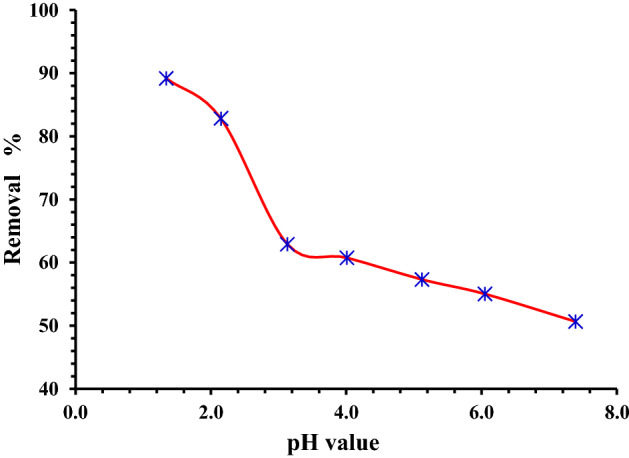
Impact of pH on the absorption of Cr^6+^ ions onto MG-OPAC (200 mg/L of Cr^6+^ ions, 2 g/L of adsorbent, 25 °C, 200 rpm, 180 min).

Because of its effect on the surface characteristics of the absorbent and Cr ion forms in water, pH is one of the most crucial factors since it regulates the absorption capacity^63,64^. Cr ions co-exist in the pH range of 1.0–6.0 in various forms, including Cr_2_O_7_^–^, HCrO_4_^–^, Cr_4_O_13_^2–^, and Cr_3_O_10_^2–^, with HCrO_4_^–^ predominating. CrO_4_^2–^ and Cr_2_O_7_^2–^ are the dominating species when the pH of the solution rises Adsorption work was accompanied by all other parameters held constant (Cr concentration = 200 mg/L; agitating speed = 200 rpm; adsorbent dose = 2.0 g/L; temperature = 25 °C) in to study the impact of solution pH on Cr^6+^ ions adsorption. The maximum amount of Cr^6+^ that could be adsorbed was 89.2 at pH 1.3. As the pH of the solution increased, the amount of chromium that could be absorbable decreased, hence (pH 1.3) was chosen as the ideal pH value for additional absorption tests (Fig. 7). Because there are more H + ions on the absorbent surface at lower pH values, there is a stronger electrostatic interaction between the positively charged absorbent surface and the chromate ions (HCrO_4_^–^). Because both anions (CrO_4_^2–^ and OH^–^) compete to be adsorbed on the surface of the absorbent, less absorption occurs at alkaline pH values^65–67^.

#### Impact of adsorbent dose and time of contact

**Figure 8:**
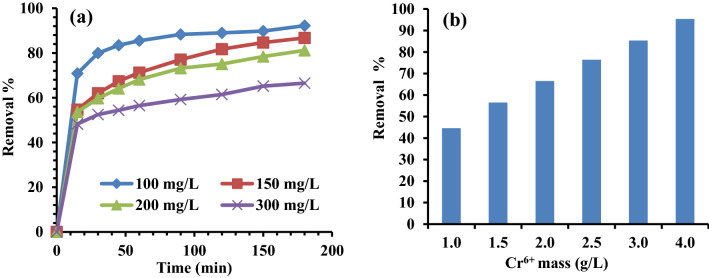
(**a**) Impact of time of contact on the absorption of varied starting concentrations of Cr^6+^ by 2 g/L of MG-OPAC, (**b**) Influence of the MG-OPAC doses on the absorption of Cr^6+^ ions, 200 rpm, 25 °C and pH 1.3.

The impact of time of contact on the absorption of diverse starting concentrations of Cr^6+^ ions (100, 150, 200, 300 mg/L) was studied using 2.0 g/L (200 mg/100 mL) of MG-OPAC at 200 rpm, 25 °C and 1.3 pH (Fig. 8a). Because it was determined from the kinetic investigation that the majority of the chromium absorption by MG-OPAC was accomplished in 180 min, these studies were carried out with a 180 min contact time.

Also, the impact of the adsorbent dose was investigated by using various adsorption doses of MG-OPAC (1, 1.5, 2, 2.5, 3, 4 g/L), 300 mg/L of Cr^6+^ ions, 200 rpm, 25 °C and 1.3 pH. The outcomes demonstrated that the percentage of Cr^6+^ adsorption increased with increasing adsorbent dose, and the highest absorption was noted with the MG-OPAC dose of 4 g/L (Fig. 8b). Increased MG-OPAC surface area and the obtainability of more absorption sites may be to blame for the rise in the percentage of absorption with absorbent dose.

#### Effect of starting Cr^6+^ ions concentration

**Figure 9:**
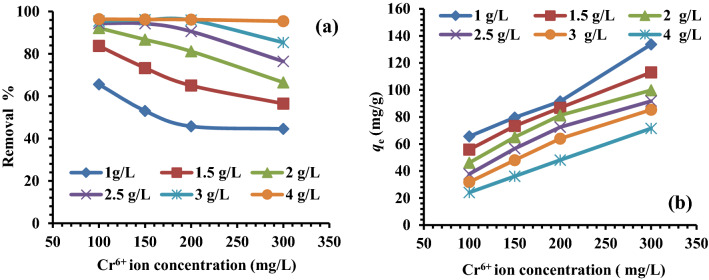
(**a**) Impact of starting Cr^6+^ ions concentration onto the removal process, (**b**) Relationship between Cr^6+^ ions absorbed quantities at equilibrium (*q*_e_) and its beginning concentration by various MG-OPAC doses.

It investigated how the starting concentration of Cr^6+^ ions in solutions affected the rate of absorption on MG-OPAC. The test solution had a pH of 1.3, adsorbent dosages of 1–4 g/L, and beginning Cr^6+^ ion concentrations of 100, 150, 200, and 300 mg/L. The experiment lasted for three hours. The findings demonstrated that when the beginning Cr concentration increased, the % chromium adsorption reduced (perhaps as a result of an increase in the mass transfer driving force) (Fig. 9a). As the concentration of Cr ions in the test solution grew, the mass of Cr absorbed per unit mass (adsorption capacity *q*_e_) of the absorbent also increased (Fig. 9b). The equilibrium absorption capacity *q*_e_ (mg/g) of the MG-OPAC was measured from Eq. (3):

3 $${q}_{e}=\frac{\left({C}_{i}-{C}_{e}\right)}{M}\times V,$$

where *C*_i_ is the beginning concentration, *C*_e_ is the equilibrium concentration, *V* is the volume of solution in liter, and *M* is the weight of the MG-OPAC. It is common practice to derive the experimental adsorption isotherms by using the absorption capacity of an absorbent determined by the mass balance on the sorbate in a system with solution volume (*V*).

### *Adsorptionisothermstudy*

The adsorption process can be estimated as a unit operation with the help of the equilibrium studies, which are crucial for optimizing the design factors of absorption systems. The solute distribution between the solid adsorbent and the liquid phase indicates the equilibrium position. To choose an appropriate model that may be applied in the design process, equilibrium data must precisely fit into various isotherm models (IMs)^68,69^. The factors achieved from the diverse isotherm models offer significant details regarding the sorbent's absorption mechanisms, surface characteristics, and empathies. Langmuir (LIM), Freundlich (FIM), and Timken (TIM) are only a few of the equations that can be used to depict equilibrium data. The applicability of IMs is matched by evaluating the *R*^2^^70^.

The fundamental tenet of the LIM is that just one absorption layer occurs following the creation of a monolayer of absorbate on the outer surface of the absorbent (without any contact between absorbed molecules)^71^. This IM also presupposes homogenous surface adsorption energies and the absence of absorbate transmigration. To estimate the maximal absorption capacity (*Q*_m_, mg/g) due to complete monolayer coverage on the MG-OPAC surface, the Langmuir isotherm model (LIM) was chosen. Equation (4) can be used to express the linear LIM.

4 $$\frac{1}{{q}_{e}}=\frac{1}{{Q}_{m}}+\frac{1}{{K}_{a}{Q}_{m}}\times \frac{1}{{C}_{e}}.$$

**Figure 10:**
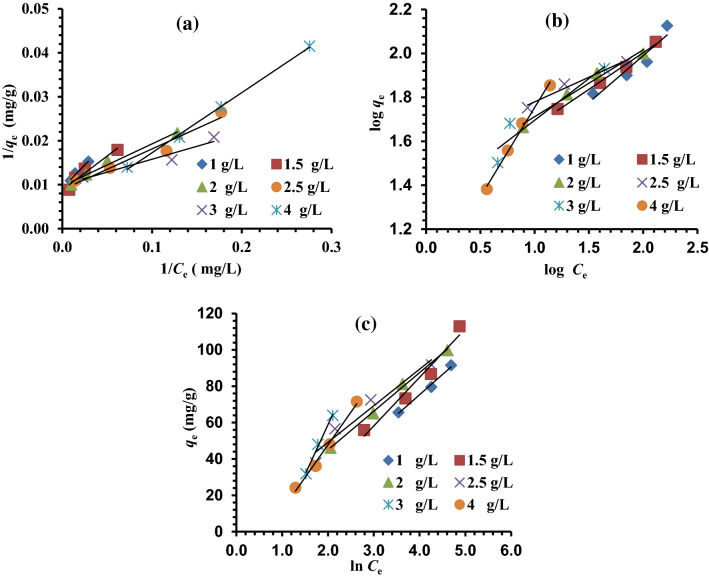
(**a**) LIM; (**b**) FIM for removal of Cr^6+^ ions; (**c**) TIM adsorption of Cr^6+^ over 1, 1.5, 2, 2.5, 3 and 4 g/L of MG-OPAC.

*K*_a_ is the LIM constant (L/mol) which is exponentially related to the heat of absorption and correlated to the absorption strength. A plot of 1/*q*_e_ versus l/*C*_e_ gives a straight line of the slope ($\frac{1}{{K}_{a}{Q}_{m}})$, and the intercept is $\frac{1}{{Q}_{m}}$ (Fig. 10a). The data obtained from LIM for the removal of Cr^6+^ ions onto MG-OPAC have *R*^2^ (0.922–0.998) (Table 2). These results indicated the fitting of LIM to the experimental adsorption results. The *Q*_m_ is 277.78 mg/g as determined by the linear solvation of LIM.

**Table 2:** LIM, FIM and TIM data for absorption of Cr^6+^ ions onto MG-OPAC (1.0–4.0 g/L).

IM	IM factors	MG-OPAC doses					
		1 g/L	1.5 g/L	2 g/L	2.5 g/L	3 g/L	4 g/L
LIM	*Q*_m_ (mg/g)	107.53	113.36	103.09	112.36	101.01	277.78
	*K*_a_ × 10^3^	44.50	56.96	101.15	96.01	168.65	26.34
	*R*^2^	0.980	0.939	0.984	0.958	0.922	0.998
FIM	1/*n*	0.43	0.33	0.30	0.22	1.18	0.81
	*K*_F_	13.71	21.67	25.56	35.92	5.50	8.71
	*R*^2^	0.905	0.992	0.981	0.974	0.971	0.992
TIM	*AT*	0.54	0.44	1.12	1.56	0.40	0.51
	*BT*	22.26	26.74	21.25	20.12	54.68	35.86
	*bT*	111.33	92.67	116.57	123.12	45.31	69.08
	*R*^2^	0.991	0.961	0.998	0.947	0.995	0.992

The first established relationship that describes the sorption process is the FIM^72,73^. The use of the FIM demonstrates that sorption energy exponentially declines on the achievement of the absorption centers of an absorbent. This FIM applies to absorption on heterogeneous surfaces with absorbed molecules interacting. Equation (5) represents the linear form of the FIM.

5 $$\mathrm{log}{q}_{e}=\mathrm{log}{K}_{F}+\frac{1}{n}\mathrm{log}{C}_{e},$$

where *K*_F_ (L/mg) is the FIM constant indicative of the comparative adsorption capacity of the adsorbent, and 1/*n* is a constant indicating the strength of sorbate absorption onto the sorbent or heterogeneity of surface, as 1/*n* value gets closer to zero, the surface becoming more heterogeneous. A value of 1/*n* < 1 indicates a normal LIM, while 1/*n* > 1 indicates cooperative absorption. A plot of log *q*_e_ versus log *C*_e_ gives a straight line with a slope of 1/*n* and an intercept of *K*_F_ (Fig. 10b). It is clear from Table 2 that the (1/*n*) values were less than 1, demonstrating that the surface nature of the MG-OPAC is heterogeneous and beneficial for the absorption process. The '*n*' value is > 1, indicating that the physical mechanism of Cr^6+^ ions adsorption onto MG-OPAC is favourable^74,75^.

According to TIM, all molecules in the layer's heat of absorption reduce linearly with coverage as a result of absorbate–absorbent interactions, and the maximal binding energy is uniformly distributed throughout the adsorption process^76,77^. The FIM equation implies that the deterioration in the absorption heat is logarithmic, whereas the TIM assumes it is linear. Equation (6) can be used to present the linear TIM.

6 $${q}_{e}=B\mathrm{ln}A+B\mathrm{ln}{C}_{e},$$

where *B* = (*RT*)/*b*, *R* is the universal gas constant, 8.314 J mol/K, and *T* is the absolute temp. in Kelvin. The constant *b* is correlated to the adsorption heat. *A* (L/g) is the TIM equilibrium binding constant in agreement with the maximum binding energy. The plot of ln *C*_e_ against *q*_e_ (Fig. 10c), the binding energy (*A*) and the isotherm constant (*b*) were measured and represented in Table 2. The Temkin isotherm appears to suit the equilibrium results for the absorption of Cr^6+^ ions onto the surface of the material (MG-OPAC) well, as seen by the correlation coefficients obtained *R*^2^ > 0.947. The results of the current study's *b* measurements for the pollutants point to some weak ionic interactions (physisorption), which confirms that the absorption is physisorption^78^.

### *Kineticstudiesofadsorption*

**Figure 11:**
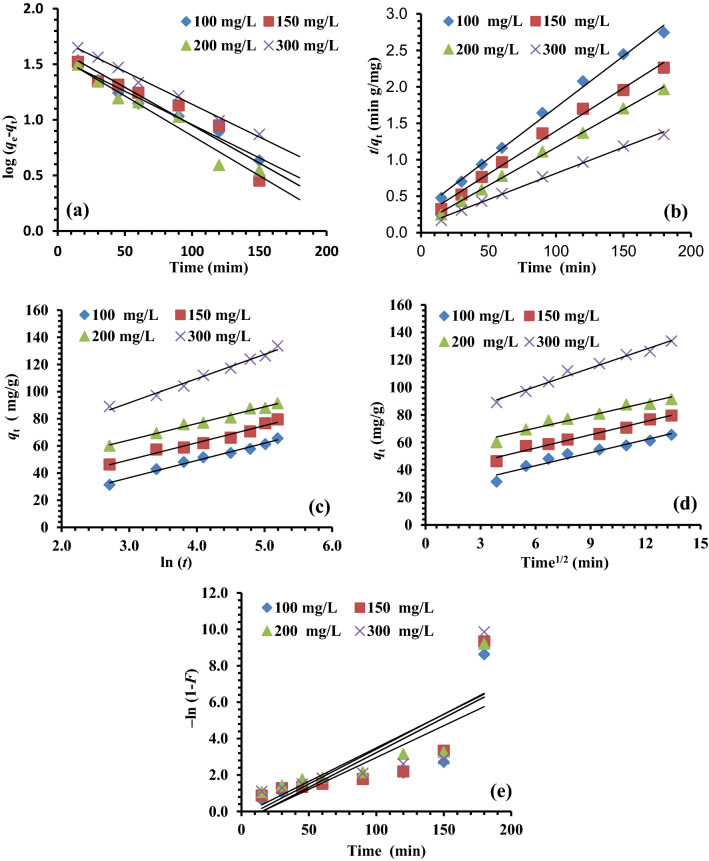
Kinetic adsorption analysis (**a**) PFOM; (**b**) PSOM, (**c**) EM, (**d**) IPDM, and (**e**) FDM.

The operating results have been fitted using kinetic models to explore the absorption mechanism, which governs the absorption process. In this study, the absorption of chromium hexavalent ions (Cr^6+^) were tested with pseudo-first-order (PFOM)^79^, pseudo-second-order (PSOM)^80^, intraparticle diffusion (IPDM)^81^, Film diffusion (FDM)^82^, and Elovich (EM) kinetic models^83–85^. The correlation coefficients (*R*^2^) represented the degree of the covenant between the model-predicted values and the work results. The PFOM is commonly conveyed by the Eq. (7) as an integrated form of PFOM.

7 $$\mathrm{log}\left({q}_{e}-{q}_{t}\right)=\mathrm{log}{q}_{e}-\frac{{k}_{1}}{2.303}t,$$

where *q*_t_ is the quantity of solute absorbed at time *t* (min), *q*_e_ is the quantity of solute (mg/g) absorbed at saturation, and *k*_1_ is the PFOM rate constant (min^−1^). The *q*_e_ and *k*_1_ values can be measured from the intercept and slope of the plots of *t* versus log (*q*_e_ − *q*_t_), respectively. The measured results obtained from the above plots were reported in Table 3. The *q*_e_ experiment does not agree with the *q*_e_ measured values and demonstrates the unsuitability of the PFOM, and the process is not a first-order reaction. The PSOM is usually expressed by the Eq. (8).

8 $$\left(\frac{t}{{q}_{t}}\right)=\frac{1}{{k}_{2}{q}_{e}^{2}}+\frac{1}{{q}_{e}}t,$$

where *q*_e_ (mg/g) is the absorbed amount at equilibrium and *K*_2_ (min/mg) is the PSOM rate constant. The plot of (*t*/*q*_t_) against (*t*) gave a straight line that was applied to measure the PSOM constants. The Cr^6+^ ions kinetics adsorption behavior onto MG-OPAC was depicted in Table 3. It is obvious from the plot results (Fig. 11b) that PSOM was best fitted with high linearity by means of *R*^2^ (> 0.99) than PFOM. Also, the *q*_e_ experimental and *q*_e_ measured results are in better covenant for the PSOM than the PFOM (Fig. 11a).

**Table 3:** Evaluation of the predicted and experimental *q*_e_ data for various starting Cr^6+^ ions and MG-OPAC doses, as well as the PFOM and PSOM adsorption rate constants.

Parameter			PFOM			PSOM			
MG-OPAC doses (g/L)	Cr^6+^ ion (mg/L)	*q*_*e*_ (exp.)	*q*_*e*_ (calc.)	*k*_*1*_ × 10^3^	*R*^*2*^	*q*_*e*_ (calc.)	*k*_*2*_ × 10^3^	*h*	*R*^*2*^
1.0	100	65.59	36.02	13.82	0.975	70.92	0.65	3245	0.995
	150	79.55	42.95	*15.66*	0.914	84.75	0.64	4598	0.992
	200	91.50	37.46	16.58	0.960	96.15	0.85	7830	0.998
	300	133.68	53.51	13.59	0.993	133.33	0.53	9462	0.997
1.5	100	55.83	24.18	19.81	0.988	58.82	1.49	5157	1.000
	150	73.30	38.02	18.42	0.936	78.74	0.80	4933	0.997
	200	86.73	39.82	17.50	0.995	91.74	0.79	6666	0.998
	300	112.97	32.97	10.13	0.975	114.94	0.84	11,049	0.995
2.0	100	46.11	9.81	15.20	0.935	46.95	3.77	8312	1.000
	150	65.06	35.24	19.58	0.983	69.93	0.92	4508	0.998
	200	81.19	35.26	16.12	0.987	85.47	0.87	6377	0.998
	300	99.79	39.46	16.81	0.908	104.17	0.86	9293	0.997
2.5	100	37.75	5.59	20.27	0.970	38.31	8.42	12,361	1.000
	150	56.57	21.63	21.65	0.989	58.82	1.96	6770	0.996
	200	72.48	43.14	20.04	0.994	78.74	0.72	4473	0.998
	300	91.72	35.55	15.66	0.963	96.15	0.88	8136	0.997
3.0	100	31.82	4.92	30.17	0.952	32.15	15.33	15,848	1.000
	150	48.04	12.84	22.80	0.979	49.26	3.89	9442	1.000
	200	63.94	21.53	23.95	0.995	66.23	2.21	9690	1.000
	300	85.35	31.51	16.58	0.990	89.29	1.06	8425	0.998
4.0	100	24.10	1.74	27.18	0.952	24.21	40.62	23,810	1.000
	150	36.10	7.28	31.55	0.992	36.63	12.12	16,260	1.000
	200	48.09	14.44	36.85	0.995	48.78	7.02	16,694	1.000
	300	71.54	23.18	20.27	0.991	74.07	4.14	22,735	1.000

The EM designates the chemisorption performance between adsorbate and adsorbent. The EM equation is the rate equation established on the absorption capacity typically provided by Eq. (9).

9 $${q}_{t}=\frac{1}{\beta }\mathrm{ln}\left(\alpha \beta \right)+\frac{1}{\beta }\mathrm{ln}(t).$$

From the plot of ln (*t*) versus *q*_t_ (Fig. 11c), the EM constants were measured from the intercept (1/*β*) ln(*αβ*) and slope (1/*β*) of the straight lines and presented in Table 4. The *R*^2^ are wavy and fluctuated between 0.865 and 0.996 without a confident role (*R*^2^ is very low), which reveals the unsuitability of EM to the work results achieved for removing Cr^6+^ ions onto MG-OPAC.

**Table 4:** The IPDM, EM, and FDM kinetic adsorption investigation results.

MG-OPAC dose (g/L)	Cr^6+^ conc	EM			IPDM			FDM		
		*β*	*α*	*R*^*2*^	*K*_*dif*_	*C*	*R*^*2*^	*K*_*FD*_	*C*	*R*^*2*^
1.0	100	0.079	1.13E + 01	0.987	3.15	24.14	0.943	0.0349	− 0.53	0.652
	150	0.078	3.34E + 01	0.969	3.21	36.67	0.975	0.0381	− 0.58	0.661
	200	0.082	1.17E + 02	0.987	3.05	52.14	0.953	0.0368	− 0.19	0.691
	300	0.056	1.53E + 02	0.985	4.53	73.35	0.984	0.0383	− 0.41	0.623
1.5	100	0.126	5.57E + 01	0.981	1.94	31.74	0.906	0.0359	− 0.03	0.768
	150	0.088	3.86E + 01	0.981	2.88	36.34	0.975	0.0362	− 0.30	0.733
	200	0.084	8.78E + 01	0.988	3.04	47.41	0.987	0.0372	− 0.28	0.706
	300	0.095	1.71E + 03	0.924	2.75	73.65	0.976	0.0338	− 0.04	0.582
2.0	100	0.25	2.97E + 03	0.952	0.95	34.16	0.859	0.0313	0.69	0.718
	150	0.10	3.69E + 01	0.996	2.52	32.84	0.983	0.0372	− 0.33	0.751
	200	0.09	8.38E + 01	0.994	2.81	44.59	0.980	0.0348	− 0.16	0.705
	300	0.09	4.73E + 02	0.974	2.81	62.46	0.993	0.0365	− 0.12	0.698
2.5	100	0.49	1.48E + 06	0.947	0.49	31.80	0.844	0.0309	1.34	0.849
	150	0.16	2.88E + 02	0.994	1.57	37.11	0.938	0.0353	0.23	0.820
	200	0.08	2.53E + 01	0.989	3.10	33.21	0.976	0.0383	− 0.45	0.753
	300	0.09	2.36E + 02	0.970	2.79	54.99	0.977	0.0359	− 0.13	0.681
3.0	100	0.80	1.14E + 09	0.925	0.30	28.30	0.812	0.0303	1.87	0.952
	150	0.23	3.49E + 03	0.966	0.99	36.11	0.852	0.0334	0.81	0.865
	200	1.59	1.08E + 02	0.991	1.54	45.22	0.909	0.0379	0.34	0.834
	300	0.10	3.36E + 02	0.994	2.45	53.47	0.986	0.0377	− 0.13	0.687
4.0	100	3.06	2.00E + 29	0.995	0.12	22.65	0.735	0.0272	2.63	0.952
	150	1.69	9.14E + 23	0.915	0.43	31.11	0.685	0.0315	1.82	0.946
	200	0.37	1.29E + 06	0.869	0.63	40.60	0.802	0.0369	1.21	0.929
	300	0.14	1.11E + 03	0.865	1.74	49.81	0.943	0.0351	0.34	0.795

Migration (transport) of the absorbate (Cr^6+^) ions from the liquid phase to the solid phase (MG-OPAC) is a step in the multi-step process of adsorption. This is followed by the diffusion of the Cr^6+^ ions into the interiors of the pores. IPDM might be the rate-regulating step in an experiment that uses a batch process and fast agitating^86,87^. This hypothesis was tested using a graph that showed the relationship between the quantities of Cr^6+^ ions absorbed (*q*_t_) and the *t*^1/2^. The IPDM is another kinetic model (KM) that should be utilized to investigate the rate-limiting phase for Cr^6+^ ions absorption onto MG-OPAC since it is likely that the Cr^6+^ ion is transferred from its water solution to MG-OPAC by IPDM. The following Eq. (10) is a typical way to express the IPDM.

10 $${q}_{t}={K}_{dif}{t}^{1/2}+C,$$

where *K*_dif_ (mg/g) is the IPDM rate constant. C (intercept) values offer evidence about the boundary layer thickness. The external mass transfer resistance increase as the intercept increase (Table 4). In Fig. 11d plots of *t*^1/2^ versus *q*_t_ result in straight lines not passed via the origin with *R*^2^ values fluctuating from low to high without confident meaning, which is revealing of some degree of boundary layer control and this additional illustration that the IPDM is not only the rate-determining step for the removal of Cr^6+^ ions by MG-OPAC but also other methods may governor the absorption rate.

The solute molecules' movement across the interface between the solid and liquid phases is crucial to adsorption^82,88,89^. Equation (11) can be used to apply the liquid film diffusion model.

11 $$\mathrm{ln}(1-F)=-{K}_{FD}(t)$$

where *K*_FD_ is the FDM rate constant and *F* is the fractional of equilibrium achievement (*F* = *q*_t_/*q*_e_). Plots of –ln (1 − *F*) against *t* (Fig. 11e), straight lines achieved did not pass via the origins, and this shows that FD is not the rate-determining step of the absorption process (Table 4).

### ***AssessmentoftheMG-OPACandotherabsorbentsforCr***^***6***+^***ionsabsorption***

To ascertain the effectiveness of the produced composite and the activation technique employed, a comparative examination of various adsorbents in expressions of absorption rate and adsorption capacity of Cr^6+^ was carried out. The findings are reported in Table 5.

**Table 5:** Assessment of various adsorbents and the MG-OPAC for the removal of Cr^6+^ ions.

Type of absorbent	Removal RATE %	*Q*_m_ (mg/g)	References
MG-OPAC	96.2	277.78	This work
*C.* *Hitosan* grafted crotonaldehyde (CGC)	98.99	434.78	^16^
Date palm seed wastes (DSC)	100	120.48	^51^
Red alga *Pterocladia* *capillacea*	58	12.85	^57^
Sugarcane bagasse	92	5.75	^90^
Maize corn-cob	62	3.0	^90^
Jatropha oil cake	97	11.75	^90^
Modified fly ashes (MFAs)	98.7	1.063	^91^
Rubber	100	43.86	^92^
Fe_3_O_4_ nanoparticles	88.83	0.610	^93^
Microporous nano-activated carbon (MNAC)	99.12	13.33	^94^
Celtek clay	–	21.55	^95^
Red algae (*Ceramium* *virgatum*)	90.0	26.5	^96^
Lichen (*Parmelina* *tiliaceae*) biomass	96.0	52.1	^97^
Moss (*Hylocomium* *splendens*) biomass	99.0	42.1	^98^
Olive Leaves	~ 52.0	42.4	^99^

### ***MechanismoftheCr***^***6***+^***ionsabsorptiononMG-OPAC***

Typically, the adsorption of metal ions involves intricate processes such as adsorption by physical forces, ion exchange, chelation, and ion entrapment in capillaries and gaps inside and between sorbent^96,100,101^. According to the FTIR study, the MG-OPAC contains several functional groups, including carboxyl, hydroxyl, and amine, which can be implicated in the binding processes. These functional groups also participate in metal ion binds, which rely on the pH level of the aqueous solution. At low pH, the positive surface charge of MG-OPAC should facilitate the binding of the negatively charged HCrO_4_^–^ ions. At the active surfaces of the MG-OPAC, acidic conditions facilitate the interchange of the HCrO_4_^–^ species with HO ions. Because the coordination interactions between metal ions and hydroxyl functional groups and other ion exchangeable moieties on the biomass surface are relatively weak in the mildly acidic solution, the sorption capacity decreased with increasing pH in the pH 3–5 range^96,102^. Furthermore, the ionized state of the MG-OPAC surface at the investigated pH is connected to the reduction in sorption at higher pH (pH > 5) in addition to the production of soluble hydroxylated complexes of the metal ions. These findings demonstrate the dominance of the pseudo-second-order sorption mechanism and the overall rate constant.

## Conclusion

This study focused on the Cr^6+^ ions adsorption from water by magnetic orange peels activated carbon (MG-OPAC). The use of an external magnetic field makes it simple to extract MG-OPAC from the medium due to its high adsorption capabilities. At pH 1.5 and 180 min of contact time, the highest level of chromium adsorption was achieved. Adsorbent dosage enhanced the adsorption percentage, with 4.0 g/L absorbent dose achieving the highest adsorption percentage. As the Cr^6+^ ions concentration grew, the adsorption percentage decreased, but the quantity of Cr^6+^ absorbed per unit mass (adsorption capacity *q*_e_) of the absorbent increased. Timken isotherm model and LIM have described the working findings well and proposed a maximum monolayer adsorption capacity (*Q*_m_) of 277.8 mg/g. The PSOM results fit well the results of absorption kinetics.

## Data Availability

The datasets used in this investigation are accessible for review upon request from the corresponding author of the paper.
